# Do hotel green practices influence customer satisfaction? Evidence from the Indian hospitality sector

**DOI:** 10.12688/f1000research.175471.1

**Published:** 2025-12-31

**Authors:** Ajit Kumar Singh, Ajay Kumar Poddar, Sumit Kumar, Mohit Dahiya, Amit Kumar Dashora, Sachin Kumar, Sandeep Paatlan, Anmol Sharma, Neetesh Bakshi, Anita Kumari Singh

**Affiliations:** 1 Chandigarh University, Mohali, Punjab, India; 2 Texila American University, Lilayi, Lusaka, Zambia; 3 Manipal Academy of Higher Education, Manipal, India; 4 University of Sunderland, Sunderland, England, UK; 5 Galgotias University, Greater Noida, Uttar Pradesh, India; 6 Panjab University, Chandigarh, Chandigarh, India; 7 Chandigarh Group of Colleges Jhanjeri, Mohali, Punjab, India

**Keywords:** Hotel green practices, customer loyalty, customer satisfaction, willingness to pay, hotel

## Abstract

**Background:**

The study investigates the impact of Hotel Green Practices (HGP) on customers’ Willingness to Pay (WTP) for the sustainable initiative, Customer Loyalty (CL), and Customer Satisfaction (CS) in the hotel industry. Although green practices have gained considerable importance in hotel operations, empirical studies that simultaneously assess their direct and mediated effects on customer satisfaction remain limited. To address this gap, the present research integrates HGP, WTP, CL, and CS into a unified structural framework and examines the mediating roles of WTP and CL in explaining consumers’ satisfaction with hotel green practices.

**Methods:**

Data were collected from the guests who stayed in star rated hotels of Delhi, India, between 9 ^th^ to 29 ^th^ October 2025. Convenience sampling was used, and a self-administered questionnaire served as the data collection tool. Measurement items were adapted from well-established scales in hospitality and consumer behaviour research. Data were analysed using SmartPLS 4, and PLS-SEM was employed to examine the structural relationships, as well as the explanatory and predictive capabilities of the proposed model.

**Results:**

The structural analysis demonstrated that HGP has a significant impact on both WTP and CL. A direct, although weaker, positive association was also observed between HGP and CS. Both mediators WTP, and CL contributed significantly to CS, with CL has the strongest effect, followed by WTP. Mediation analysis confirmed partial mediation, with indirect effects surpassing the direct effect of HGP on CS. The model showed substantial explanatory power, particularly for CS (R ^2^ = 0.627), and demonstrated strong predictive relevance, with Q ^2^ values exceeding the recommended threshold of 0.35.

**Conclusions:**

The findings suggest that HGP has positive impact on CS, CL, and customers’ WTP. Furthermore, customer loyalty emerged as a strong mediating variable between hotel green practices and customer satisfaction relationship. Overall, these results highlight the importance of adopting sustainability practices in hotel operations, as they not only strengthen customer loyalty and customer satisfaction, but also influence customers to pay premium price for the hotel’s sustainable initiatives.

## 1. Introduction

Greenhouse emissions, waste production, and environmental pollution has become major issues in the hotel operation ( Bohdanowicz, 2006; Gössling, 2015). Thus, hotels now a days are heavily using eco-friendly practices in their daily activities ( Han & Yoon, 2015; Mensah, 2006). According to Han et al. (2019) and Chang et al. (2024), hotel green practices involves adoption of green initiatives and socially responsible initiatives in hotel operations. These practices has not only influences environmental concerns positively, but has also improves customer satisfaction significantly ( Genç & Zengin, 2025). Several studies also confirm that green initiatives positively influence customer satisfaction, attitudes, and behavior ( Han et al., 2010; Kang et al., 2012; Rahman & Reynolds, 2016; Han & Hyun, 2018; Kandampully & Suhartanto, 2000). These activities also influence brand image ( Chen, 2010; Rahman and Reynolds, 2016), trust and brand equity ( Chen, 2010) significantly. Han et al. (2010) concluded that eco-friendly operations have an important influence on hotel choice intentions. Kang et al. (2012) found that consumers are ready to pay a high price when a hotel conducts genuine environmental activities.

Most existing studies have examined the impact of green hotel practices on customer satisfaction ( Han et al., 2010; Han & Hyun, 2018), and most of these studies were conducted in developing nations ( Chen, 2010; Han et al., 2010; Rahman and Reynolds, 2016). However, developing markets such as India are emerging in tourism, and consumer expectations remain undiscovered. In addition, mediators, including financial mediators, such as willingness to pay, and relational mediators, such as customer loyalty, are often examined independently and not in an integrated system.

The study area considered for this empirical study is Delhi, India, and its sustainability challenges ( Singh et al., 2014) provide a particularly relevant context for this investigation. Addressing these gaps, the present study investigates the direct and indirect relationship between HGP, CS, and proposes below research questions:

***RQ1:** Do hotel green practices (HGP) influence customer satisfaction?*

***RQ2:** Do customers’ willingness to pay and customer loyalty mediate the relationship between Hotel Green Practices and customer satisfaction?*

## 2. Literature review

### 2.1 Hotel green practices and willingness to pay

In the hotel industry, sustainable practices benefit all internal and external stakeholders, as well as long-term reputational and financial results ( Font et al., 2016; Yusof et al., 2022). Many studies have advocated the importance of technology adoption, sustainable practices, ecotourism awareness, and customer preferences in the hotel industry ( Kaurav et al., 2020; Baber et al., 2015; Rana et al., 2023; Raina et al., 2024). Consumers are also becoming eco- friendly now a days, and are willing to pay premium price for the environmentally friendly products ( Kang et al., 2012; Damigos, 2023). Many studies advocates that hotel green initiatives positively affect customers’ WTP because of their visibility and credibility ( Rahman and Reynolds, 2019; Barber, 2014). Kang et al. (2012) established that customers of hotels in the U.S. were ready to pay up to 6% more in eco-certified hotels. This trend was confirmed by Damigos (2023), who conducted a global systematic review and found that tourists are willing to pay a premium price to stay in sustainable accommodations. Similarly, Han et al. (2019) reveal that guests’ perceived environmental responsibility increases their readiness to provide financial assistance to hotels’ green efforts. The implications of these findings are that the perceived value of hotel services improves because green practices are implemented and communicated effectively, thereby improving guests’ WTP.

H1:
*HGP has a positive impact on customers’ WTP.*

### 2.2 Customer loyalty and green hotel practices

Customer loyalty refers to long-term attitudinal and behavioral loyalty to a service provider ( Oliver, 1999). Service quality, trust, and emotional attachment to the brand determine loyalty in hospitality ( Bowen and Chen, 2001). Green practices are also instrumental in creating a feeling of loyalty by generating value congruence between the personal values of the customer and the environmental ethics of hotels ( Genç and Zengin, 2025). Moise et al. (2018) found that guests who view hotels as environmentally responsible have increased brand loyalty and positive word-of-mouth. Han, Yu, and Kim (2019) also highlighted that eco-friendly hotels significantly impacts customer trust, which results in attitudinal and behavioural loyalty.

H2:
*HGP has a positive impact on CL.*

### 2.3 Customer satisfaction and hotel green practices

Customer satisfaction is the cumulative judgment of a service encounter in comparison to anticipations ( Oliver, 1980). Regarding green hotels, satisfaction does not solely depend on the traditional service quality aspects, but on the perceived performance in the environmental aspects as well ( Soni et al., 2022; Chang et al., 2024). The empirical data show that the guests gain satisfaction when they find green practices of hotels to be genuine and close to sustainable values ( Rahman and Reynolds, 2019). The study of Chang et al. (2024) discovered that green initiatives have a positive impact on visitor satisfaction and revisit intention. Similarly, Moise et al. (2018) found that green initiative influence customer loyalty and customers’ willingness to pay.

H3:
*HGP positively impact CS.*

### 2.4 Willingness to pay and customer satisfaction

When guests are more willing to pay for sustainable services, they tend to be more satisfied because they are contributing to the services according to their moral and environmental beliefs ( Han and Hyun, 2017) revealed that eco-friendly consumers experience a sense of psychological fulfilment when they patronize green hotels, which increases their satisfaction. Damigos (2023) also advocates that WTP mediates the connection between perceived sustainability and tourism consumption satisfaction. This implies that guests’ readiness to spend on green programs reflects both altruistic and hedonic motivations, which add to satisfaction.

H4:
*WTP positively impact CS.*

### 2.5 Customer satisfaction and customer loyalty

Satisfaction is a major antecedent of loyalty in hospitality, but reciprocal causality is also found i.e., satisfied customers are likely to express greater satisfaction because they have a positive experience and accumulate it ( Bowen and Chen, 2001). The empirical data support positive relationship between customers’ satisfaction and loyalty. Moise et al. (2018) advocates that the loyal guests has higher satisfaction due to the constant fulfilment of their expectations. Han and Hyun (2017) also reveals that loyalty is a positive reinforcement of satisfaction by creating emotional attachment to the brand.

*H5*: *CS positively impact CL.*

### 2.6 Mediating roles of WTP and CL

Indirect linkages between HGP and CS through WTP and CL have been established in sustainability studies. Han et al. (2019) found that the guests’ willingness to pay for eco-friendly services mediates the effect of green practices on satisfaction. Genç & Zengin (2025) reported that loyalty serves as a critical conduit through which environmental responsibility enhances satisfaction. Furthermore, mediating effects highlight psychological mechanisms by which guests translate green perceptions into satisfaction. WTP reflects cognitive evaluation of value, while CL embodies affective commitment ( Oliver, 1999). Integrating both mediators provides a holistic understanding of how green practices ultimately improve satisfaction.

H6:
*WTP significantly mediates the relationship between HGP and CS.*

H7:
*CL significantly mediates the relationship between HGP and CS.*

**Figure 1.**
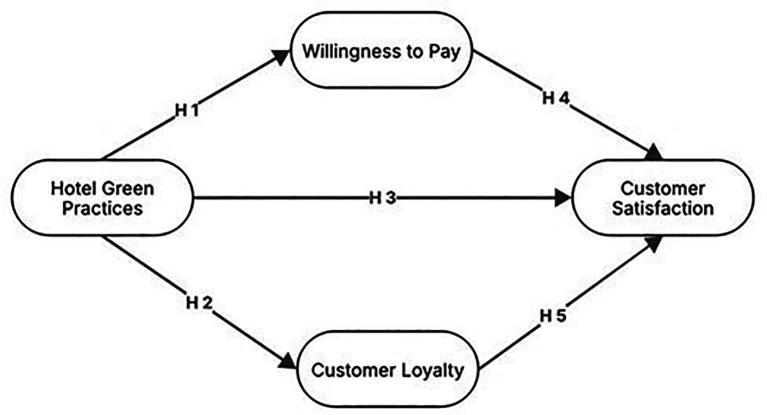
Proposed theoretical framework.

Based on the above arguments, the conceptual framework proposed for the study is presented in Figure 1.

## 3. Research methodology

The study used a cross-sectional design and Partial Least Squares Structural Equation Modeling (PLS-SEM) is proposed to test the measurement and structural model. Data were collected using a convenience sampling method, and all respondents were aged 18 years or older. Written informed consent was obtained from all participants prior to their participation in the study. Participants were informed about the study objectives, their right to withdraw at any stage, and the measures taken to ensure the confidentiality and anonymity of their responses. Between 9 ^th^ and 29 ^th^ October 2025, individuals staying in star-rated hotels in Delhi were approached with a self-administered survey instrument. A 5-point Likert scale was employed for all items (1 = strongly disagree, 5 = strongly agree). From the 510 questionnaires distributed, 79 were excluded due to incomplete or invalid responses, resulting in 431 usable questionnaires for the analysis. The HGP construct was evaluated using four items ( Han, Hsu, & Sheu, 2010), WTP with three items derived from ( Namkung & Jang, 2013), CL through four items ( Zeithaml, Berry, & Parasuraman, 1996; Prayag & Ryan, 2012), and CS was measured through four items ( Merli et al., 2018; Oliver, 1996). Details of constructs are their items are presented in Table 1.

**Table 1.** Details of constructs and their items.

Hotel Green Practices (HGP): Adapted from Han, Hsu, & Sheu (2010)	
HGP1	This hotel makes substantial efforts to reduce energy consumption (e.g., LED lighting, smart thermostats).
HGP2	This hotel has an active program to reduce water use (e.g., low-flow faucets, linen reuse).
HGP3	This hotel minimizes waste by recycling and composting.
HGP4	This hotel offers environmentally friendly guest amenities (e.g., bulk dispensers, eco-certified toiletries).
*Willingness to Pay (WTP): Adapted from* *Namkung & Jang (2013)*	
WTP1	I am willing to pay a higher price for a room in this green hotel compared to a regular one.
WTP2	I would accept higher room rates if environmentally friendly practices are guaranteed.
WTP3	I would pay more, if necessary, to stay in a hotel that is environmentally responsible.
*Customer Loyalty (CL): Adapted from* *Zeithaml, Berry, & Parasuraman (1996)* *; widely applied in hotel studies such as* *Prayag & Ryan (2012)*	
CL1	I intend to stay in this hotel again in the future.
CL2	I will recommend this hotel to friends and relatives.
CL3	If I need accommodation in this area, this hotel would be my first choice.
CL4	I would say positive things about this hotel to others.
*Customer Satisfaction (CS): Adapted from* *Merli et al. (2018)* *and* *Oliver (1996)*	
CS1	I am happy with my decision to stay here because of its environmental practices.
CS2	I am pleased with the hotel’s eco-friendly products/services.
CS3	Overall, I feel satisfied with the hotel’s environmental performance.
CS4	My decision to purchase this hotel’s services was influenced by its green image.

Source: Author’s computation.

### 3.1 Measurement model

**Figure 2.**
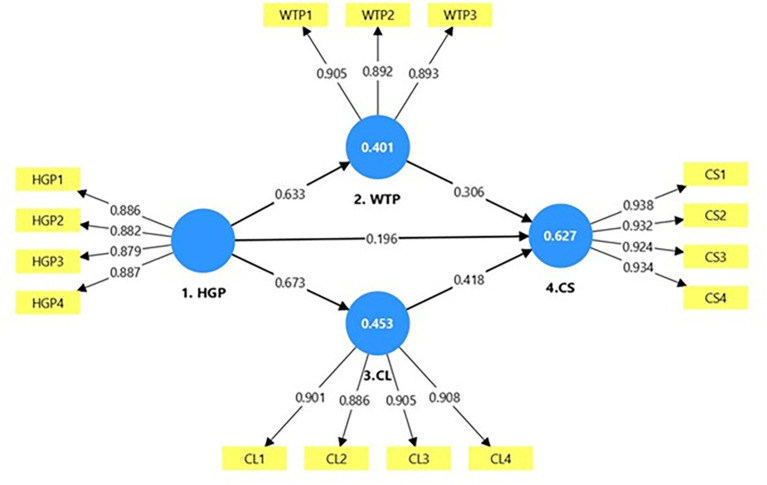
Measurement model.

As shown in Figure 2, the measurement model presents the outer and inner loadings for the four latent variables HGP, WTP, CL, and CS. Table 2 summarizes the results of all reliability statistics used in this study. All constructs reported Cronbach’s alpha values exceeding the recommended threshold of 0.70, confirming satisfactory reliability. The rho_A values were more than 0.70, and positioned between Cronbach’s alpha and ρ_C. As shown in Table 2, all AVE values were greater than 0.50, thus supporting convergent validity ( Fornell & Larcker, 1981).

**Table 2.** Factor loadings, reliability and validity.

	Factors loading	Cronbach's alpha	Composite reliability (rho_a)	Composite reliability (rho_c)	Average variance extracted (AVE)	VIF
Customer Loyalty		0.922	0.923	0.945	0.811	
CL1	0.901					3.026
CL2	0.886					2.78
CL3	0.905					3.196
CL4	0.908					3.298
Customer Satisfaction		0.95	0.95	0.964	0.869	
CS1	0.938					4.814
CS2	0.932					4.338
CS3	0.924					3.996
CS4	0.934					4.633
Hotel Green Practices		0.907	0.907	0.935	0.781	
HGP1	0.886					2.682
HGP2	0.882					2.653
HGP3	0.879					2.603
HGP4	0.887					2.721
Willingness to Pay		0.878	0.879	0.925	0.804	
WTP1	0.905					2.499
WTP2	0.892					2.331
WTP3	0.893					2.4

Source: Author’s computation.

The cross-loading results revealed that each item loaded more strongly on its intended construct than on all other constructs. The HTMT values were all below the conservative threshold (<0.85). Table 4 summarizes the Fornell–Larcker statistics, showing that the square roots of AVEs for all constructs exceeded their inter construct correlations, thereby confirming discriminant validity ( Fornell & Larcker, 1981). Furthermore, the HTMT results presented in Table 3 were below the conservative threshold of 0.85, further supporting construct distinctiveness ( Henseler, Ringle, & Sarstedt, 2015). Together, these results indicate that HGP, WTP, CL, and CS are distinct from one another.

**Table 3.** Discriminant validity-HTMT–test.

	1. HGP	2. WTP	3. CL	4. CS
1. HGP				
2. WTP	0.709			
3. CL	0.736	0.585		
4. CS	0.723	0.711	0.759	

Source: Author’s computation.

**Table 4.** Discriminant validity Fornell Lacker – Statistics.

	1. HGP	2. WTP	3. CL	4. CS
HGP	**0.884**			
WTP	0.633	**0.897**		
CL	0.673	0.527	**0.9**	
CS	0.671	0.65	0.711	**0.932**

Source: Author’s computation.

Finally, multicollinearity among predictors was evaluated through variance inflation factor (VIF) statistics ( Table 2). All VIF values were well below 5, suggesting that multicollinearity is not a concern in this model ( Kock, 2015; Diamantopoulos & Siguaw, 2006).

### 3.2 Structural model evaluation and hypothesis testing

**Figure 3.**
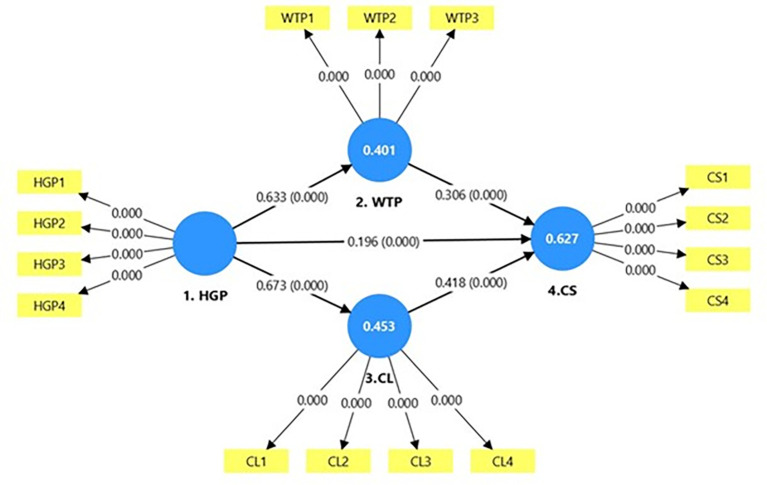
Structural model.

Figure 3 presents the structural model of the proposed study. HGP had a significant positive impact on both WTP (β = 0.633, t = 20.715, p < 0.001) and CL (β = 0.673, t = 25.670, p < 0.001), indicating that sustainable initiatives increase guests’ willingness to pay and strengthen loyalty. H3 confirmed significant, and positive direct relationship between HGP and CS (β = 0.196, t = 4.495, p < 0.01), suggesting that green practices influence satisfaction primarily through mediating mechanisms. H4 confirmed that WTP positively affects CS (β = 0.306, t = 7.838, p < 0.001), while H5 showed that CL has the strongest impact on CS (β = 0.418, t = 10.651, p < 0.001). These results highlight loyalty as a key driver of satisfaction in green hospitality settings. Each of these findings is summarized in Table 5.

**Table 5.** Path coefficient – Hypothesis testing.

	Original sample	Standard deviation	T statistics	P values	Result
H1: HGP -> WTP	0.633	0.031	20.715	0	Accepted
H2: HGP -> CL	0.673	0.026	25.67	0	Accepted
H3: HGP -> CS	0.196	0.044	4.495	0	Accepted
H4: WTP -> CS	0.306	0.039	7.838	0	Accepted
H5: CL -> CS	0.418	0.039	10.651	0	Accepted

Source: Author’s computation.

Mediation analyses were conducted to examine the mediating roles proposed in H6 and H7. For H6 examined whether WTP mediates the relationship between HGP and CS. The results (see Table 6) indicated a significant indirect effect of HGP on CS through WTP (β = .193, t = 7.031, p < .001). The total effect of HGP on CS remained significant (β = .671, t = 25.283, p < .001), and after the inclusion of mediator (WTP), the direct effect of HGP on CS also remained significant (β = .196, t = 4.495, p < .001), demonstrating a complementary partial mediation and thereby supporting H6. Similarly, H7 proposed that CL mediates the relationship between HGP and CS. The findings revealed a significant indirect effect of HGP on CS through CL (β = .282, t = 9.564, p < .001). The total effect of HGP on CS was again significant (β = .671, t = 25.283, p < .001), and with the presence of mediator (CL), the direct effect of HGP on CS remained significant (β = .196, t = 4.495, p < .001), indicating a complementary partial mediation.

**Table 6.** Mediation analysis.

	Total effect		Direct effect		Specific indirect effect			
	β	p-value	B	p-value	β	t-value	p-value	Results
H6: HGP→ WTP → CS	0.671	0	0.196	0	0.193	7.031	0	Partial Mediation
H7: HGP → CL → CS	0.671	0	0.196	0	0.282	9.564	0	Partial Mediation

Source: Author’s computation.

Table 7, presents model’s explanatory power. HGP explains 40.1% of the variance in WTP (R ^2^ = 0.401) and 45.3% of the variance in CL (R ^2^ = 0.453). Customer Satisfaction (CS) has the highest explanatory power, with an R ^2^ of 0.627, indicating that HGP, WTP, and CL together explain nearly 63% of its variance. The adjusted R ^2^ (see Table 8) values for WTP (0.399), CL (0.452), and CS (0.625) closely align with their respective R ^2^ values, confirming the model’s robustness and absence of overfitting.

**Table 7.** r Square.

	Original sample	Sample mean	Standard deviation	T statistics	P values
WTP	0.401	0.403	0.039	10.382	0
CL	0.453	0.454	0.035	12.874	0
CS	0.627	0.63	0.028	22.581	0

Source: Author’s computation.

**Table 8.** Adjusted r square.

	Original sample	Sample mean	Standard deviation	T statistics	P values
2. WTP	0.399	0.401	0.039	10.322	0
3. CL	0.452	0.453	0.035	12.808	0
4. CS	0.625	0.627	0.028	22.33	0

Source: Author’s computation.

Effect size results in Table 9 further clarify the strength of these relationships. HGP shows a large effect on both WTP (f ^2^ = 0.669) and CL (f ^2^ = 0.829), demonstrating that sustainability practices strongly influence willingness to pay and loyalty. In contrast, HGP’s direct effect on CS is small (f ^2^ = 0.045), indicating that its impact on satisfaction operates mainly through the mediating roles of WTP and CL. Overall, these findings highlight the importance of integrating green practices with value-driven mechanisms to better predict customer satisfaction in sustainable hospitality settings.

**Table 9.** f Square.

	Original sample	Sample mean	Standard deviation	T statistics	P values
1. HGP -> 2. WTP	0.669	0.682	0.11	6.091	0
1. HGP -> 3. CL	0.829	0.841	0.12	6.932	0
1. HGP -> 4. CS	0.045	0.048	0.022	2.096	0.018
2. WTP -> 4. CS	0.145	0.149	0.04	3.664	0
3. CL -> 4. CS	0.249	0.253	0.052	4.744	0

Source: Author’s computation.

Table 9 reported the effect sizes of WTP and CL. The result revels that both WTP and CL significantly contribute to customer satisfaction, with CL (f ^2^ = 0.249) exerting a slightly stronger influence than WTP (f ^2^ = 0.145). Table 10 summarized the result of predictive relevance. The Q ^2^ values for WTP (0.397), CL (0.451), and CS (0.448) exceeds the threshold value of 0.35. These results indicate strong out-of-sample predictive capability. Additionally, the PLS Predict results reported in Table 11 show that the PLS model demonstrates lower or comparable prediction errors relative to linear regression benchmarks, confirming its high predictive accuracy.

**Table 10.** PLS predict MV summary.

	Q ^2^predict	PLS-SEM_RMSE	PLS-SEM_MAE	LM_RMSE	LM_MAE	IA_RMSE	IA_MAE
WTP1	0.351	0.791	0.624	0.797	0.63	0.982	0.708
WTP2	0.309	0.756	0.602	0.757	0.603	0.909	0.659
WTP3	0.296	0.755	0.591	0.76	0.595	0.899	0.658
CL1	0.397	0.742	0.602	0.746	0.606	0.955	0.715
CL2	0.338	0.772	0.631	0.776	0.634	0.949	0.713
CL3	0.373	0.796	0.621	0.802	0.626	1.005	0.738
CL4	0.351	0.77	0.627	0.775	0.631	0.956	0.704
CS1	0.397	0.863	0.707	0.868	0.708	1.111	0.852
CS2	0.389	0.915	0.744	0.921	0.747	1.17	0.931
CS3	0.399	0.808	0.658	0.813	0.662	1.043	0.791
CS4	0.369	0.915	0.745	0.921	0.753	1.152	0.887

Source: Author’s computation.

**Table 11.** PLS predict LV summary.

	Q ^2^predict	RMSE	MAE
2. WTP	0.397	0.78	0.604
3. CL	0.451	0.744	0.594
4. CS	0.448	0.746	0.605

Source: Author’s computation.

## 4. Discussion

### 4.1 Theoretical implications

The study concludes that HGP significantly impacts CS, WTP and CL. Similar, results have been reported in previous studies, showing that green practices influences customer attitudes and behavioural intentions ( Chen, 2010; Han et al., 2010; Kang et al., 2012). The path coefficient between the HGP and CL linkage is significantly high, which supports that sustainable practices can build a strong relation between hotels and their guests. Hotel green practices positively influence environmentally mindful guests, which further results in increase in emotional bonding, word of mouth, and customer loyalty towards the brand ( Han and Hyun, 2018). This will not only significantly increase repeat business but also cut acquisition costs. The study also reveals that the impact of HGP on CS is comparatively low as compared to the mediating variables i.e, WTP and CL. This finding suggest that along with hotel green practices, WTP and CL also plays significant role for the customer satisfaction. In other words guest satisfaction increases, when HGP coupled with WTP and CL. By integrating WTP and CL as a mediating variables, and within a single structural model, the study offers a more holistic explanation of how green practices influence consumer satisfaction in hospitality settings. The empirical results further revels that the model has strong explanatory power (R ^2^ = 0.627) and predictive relevance (Q ^2^ > 0.35), confirming its theoretical soundness and empirical reliability ( Chin, 1998; Hair et al., 2019; Shmueli et al., 2019). Overall, results indicate that Indian hotel customers respond positively to hotel green initiatives, reflecting a broader shift toward more environmentally responsible consumption patterns.

### 4.2 Practical implications

From a hotels perspective, the results have a number of actionable insights. First, hotel operators should strategically invest in and communicate green practices, not merely as compliance with environmental regulations but as mechanisms to enhance brand loyalty and willingness to pay. The installation of energy-saving systems, the adoption of water saving technologies, complete separation of waste will attract environmentally conscious customers. The customers’ trust on a hotel brand can be strengthen, when hotel clearly mention their green certifications (e.g., LEED, Green Key) or provide real-time sustainability data (e.g., energy-saving dashboard). Second, there is a need to have a communication policy to customers to translate the green practices into economic and relationship terms. The willingness to engage in environmental activities, be it on websites, in-room, mobile applications, and even in communication with the staff, may be the defining factor of whether the guests pay a higher rate ( Kang et al., 2012). Moreover, loyalty could be reinforced and sustainable behavior promoted by green loyalty programmes which reward environmentally friendly behavior (e.g., more points on reusing towels, using digital check-out, or tree-planting programmes). Third, the effective implementation of green initiatives depends on the training and engagement of the staff. The ambassadors of the environmental initiatives of the hotel will be frontline workers, and by offering them knowledge and incentives to promote green initiatives, the overall experience and satisfaction of the guests can be enhanced. The results are educative to the government agencies, tourism boards and industrial associations. Fiscal incentives (e.g., tax exemption of certified properties), easy access to green finance, and certification are one of the ways of persuading hotels to adopt green practices. Regulatory regimes can also be used to improve consumer trust by ensuring that sustainability indicators are clearly reported. In addition, special awareness campaigns should be used to sensitize local and foreign tourists to the environmental effect of their choice of accommodation, which will increase the demand for green hotels. Industry associations can assist hotels in sharing knowledge, capacity-building programs, and joint marketing of certified green hotels as part of India’s sustainable tourism positioning.

## 5. Conclusion

This study provides strong empirical data on the effect of hotel green practices on customer satisfaction, which is manifested in the willingness to pay and loyalty towards the customer in the case of star-rated hotels in Delhi, India. This study improves the theoretical understanding of the processes by which sustainability and customer outcomes are connected by developing and confirming a dual-mediation model using PLS-SEM. The results show that hotel green practices have a direct influence on satisfaction, but indirect effects through loyalty and willingness to pay are stronger, with the former being the most significant channel. This highlights that sustainable initiatives generate both operational benefits and emotional value, which are essential for customer satisfaction and competitive advantage. The study contributes theoretically by integrating environmental factors with relationship marketing and value perspectives into a unified framework. It also offers practical evidence for hoteliers and policymakers seeking to align sustainability objectives with business performance. Incorporating environmental practices into service delivery and relationship-building strategies can enable hotels to increase loyalty, justify premium rates, and boost overall guest satisfaction. Therefore, this study confirms that sustainability and profitability are not conflicting but complementary goals.

## 6. Limitations and future research

First, only five star hotels in Delhi were used as the sample, which can limit the extrapolation of the results. Customer’s attitudes and behaviours toward green practices will most likely differ by geographic area, culture, and hotel typologies. Future efforts should aim to duplicate the current research in other cities or countries to strengthen external validity and explore the possibility of cross-cultural variations in the mediating processes. Second, causal inference is restricted because of the use of cross-sectional survey data. Despite the theoretical soundness of the structural model, a longitudinal or experimental design might have been more suitable to grasp the dynamics of time in terms of green practices, loyalty, willingness to pay, and satisfaction. Third, this study focused on customer loyalty and willingness to pay as the mediating variables of interest. These relationships could also be mediated or moderated by additional psychological variables, including perceived authenticity, environmental involvement, trust, or green brand image ( Chen, 2010). These constructs should be included in future models to provide a more comprehensive insight into how customers respond to green practices. Finally, although this study was customer-centred, a multi-stakeholder approach that includes the involvement of employees, managerial intentions, and policy frameworks could be used in future studies.

## Ethics statement

The research protocol was reviewed and approved by the Ethics Committee of Texila American University, Zambia (Approval No: TAUZ/REC/2025/S/45; 15 September 2025).

## Data Availability

The dataset and questionnaire employed in this study is openly available on Zenodo (Data set: https://doi.org/10.5281/zenodo.17848960; Questionnaire: https://doi.org/10.5281/zenodo.17848885) ( Singh, 2025a; Singh, 2025b). All data are provided under the International License CC BY 4.0.

## References

[ref1] Baber R Kaurav RPS Williams RL Jr : How travelers differ in their preferences regarding hotel selection: Empirical evidence from travelers in India. *Asian Journal of Tourism and Hospitality Research.* 2015;8(1):15–26.

[ref2] Barber NA : Profiling the potential “green” hotel guest: Who are they and what do they want? *J. Hosp. Tour. Res.* 2014;38(3):361–387. 10.1177/1096348012451462

[ref3] Bohdanowicz P : Environmental awareness and initiatives in the Swedish and Polish hotel industries—Survey results. *Int. J. Hosp. Manag.* 2006;25(4):662–682. 10.1016/j.ijhm.2005.06.006

[ref4] Bowen JT Chen SL : The relationship between customer loyalty and customer satisfaction. *Int. J. Contemp. Hosp. Manag.* 2001;13(5):213–217. 10.1108/09596110110395893

[ref5] Chang R Ho C Ho S : Assessing green practices on eco-friendly hotels: Effects on visitor satisfaction, revisit intention, premium willingness to pay and word-of-mouth. *Sustainability.* 2024;16(9). 10.3390/su16093834

[ref6] Chen Y-S : The drivers of green brand equity: Green brand image, green satisfaction, and green trust. *J. Bus. Ethics.* 2010;93(2):307–319. 10.1007/s10551-009-0223-9

[ref7] Chin WW : The partial least squares approach to structural equation modeling. Marcoulides GA , editor. *Modern methods for business research.* Lawrence Erlbaum Associates;1998; pp.295–336.

[ref8] Damigos D : How much are consumers willing to pay for a greener hotel industry? A systematic literature review. *Sustainability.* 2023;15(11):8775. 10.3390/su15118775

[ref9] Diamantopoulos A Siguaw JA : Formative versus reflective indicators in organizational research: A comparison and evaluation. *Br. J. Manag.* 2006;17:263–282. 10.1111/j.1467-8551.2006.00500.x

[ref10] Font X Elgammal I Lamond I : Greenhushing: The deliberate under-communication of sustainability practices by tourism businesses. *J. Sustain. Tour.* 2016;25(7):1007–1023. 10.1080/09669582.2016.1158829

[ref11] Fornell C Larcker DF : Evaluating structural equation models with unobservable variables and measurement error. *J. Mark. Res.* 1981;18(1):39–50. 10.1177/002224378101800104

[ref12] Genç G Zengin B : How do green hotel practices affect guests’ behavioral intentions? A PLS-SEM approach. *Tourism Management Studies.* 2025;21:1–16. (Special issue). 10.18089/tms.2025.03.01

[ref13] Gössling S : New performance indicators for water management in tourism. *Tour. Manag.* 2015;46:233–244. 10.1016/j.tourman.2014.06.018

[ref14] Hair JF Hult GTM Ringle CM : *A primer on partial least squares structural equation modeling (PLS-SEM).* SAGE Publications; 2nd ed. 2019.

[ref15] Han H Hyun SS : Impact of hotel-restaurant image and quality of physical-environment, service, and food on satisfaction and intention. *Int. J. Hosp. Manag.* 2017;63:82–92. 10.1016/j.ijhm.2017.03.006

[ref16] Han H Hyun SS : Role of motivations for luxury cruise traveling, satisfaction, and involvement in building traveler loyalty. *Int. J. Hosp. Manag.* 2018;70:75–84. 10.1016/j.ijhm.2017.10.024

[ref17] Han H Yoon HJ : Hotel customers’ environmentally responsible behavioral intention: Impact of key constructs on decision in green consumerism. *Int. J. Hosp. Manag.* 2015;45:22–33. 10.1016/j.ijhm.2014.11.004

[ref44] Han H Hsu L-TJ Sheu C : Application of the theory of planned behavior to green hotel choice: Testing the effect of environmental friendly activities. *Tour. Manag.* 2010;31(3):325–334. 10.1016/j.tourman.2009.03.013

[ref19] Han H Yu J Kim W : Environmental corporate social responsibility and the strategy to boost the airline’s image and customer loyalty intentions. *J. Travel Tour. Mark.* 2019;36(3):371–383. 10.1080/10548408.2018.1557580

[ref20] Henseler J Ringle CM Sarstedt M : A new criterion for assessing discriminant validity in variance-based structural equation modeling. *J. Acad. Mark. Sci.* 2015;43:115–135. 10.1007/s11747-014-0403-8

[ref21] Kandampully J Suhartanto D : Customer loyalty in the hotel industry: The role of customer satisfaction and image. *Int. J. Contemp. Hosp. Manag.* 2000;12(6):346–351. 10.1108/09596110010342559

[ref22] Kang KH Stein L Heo CY : Consumers’ willingness to pay for green initiatives of the hotel industry. *Int. J. Hosp. Manag.* 2012;31(2):564–572. 10.1016/j.ijhm.2011.08.001

[ref23] Kaurav RPS Baber R Rajput S : *Technology-driven tourism and hospitality industry as a tool for economic development: A bibliometric analysis* *The Emerald handbook of ICT in tourism and hospitality.* Emerald Publishing Limited;2020; pp.469–486. 10.1108/978-1-83982-688-720201030

[ref24] Kock N : Common method bias in PLS-SEM: A full collinearity assessment approach. *International Journal of e-Collaboration (ijec).* 2015;11(4):1–10. 10.4018/ijec.2015100101

[ref25] Mensah I : Environmental management practices among hotels in the greater Accra region. *Int. J. Hosp. Manag.* 2006;25(3):414–431. 10.1016/j.ijhm.2005.02.003

[ref26] Merli R Preziosi M Acampora A : Why should hotels go green? Insights from guests experience in green hotels. *Int. J. Hosp. Manag.* 2018;81:169–179. 10.1016/j.ijhm.2019.04.022

[ref27] Moise M-S Gil-Saura I Ruiz-Molina M-E : Effects of green practices on guest satisfaction and loyalty. *European Journal of Tourism Research.* 2018;20:92–104. 10.54055/ejtr.v20i.342

[ref28] Namkung Y Jang S : Effects of restaurant green practices on brand equity formation: Do green practices really matter? *Int. J. Hosp. Manag.* 2013;33:85–95. 10.1016/j.ijhm.2012.06.006

[ref29] Oliver RL : A cognitive model of the antecedents and consequences of satisfaction decisions. *J. Mark. Res.* 1980;17(4):460–469. 10.1177/002224378001700405

[ref30] Oliver RL : *Satisfaction: A behavioral perspective on the consumer.* McGraw-Hill;1996.

[ref31] Oliver RL : Whence consumer loyalty? *J. Mark.* 1999;63(Special Issue):33–44. 10.1177/00222429990634s105

[ref32] Prayag G Ryan C : Antecedents of tourists’ loyalty to Mauritius: The role and influence of destination image, place attachment, personal involvement, and satisfaction. *J. Travel Res.* 2012;51(3):342–356. 10.1177/0047287511410321

[ref33] Rahman I Reynolds D : Predicting green hotel behavioral intentions using a theory of environmental commitment and sacrifice for the environment. *Int. J. Hosp. Manag.* 2016;52:107–116. 10.1016/j.ijhm.2015.09.007

[ref34] Rahman I Reynolds D : The influence of values and attitudes on green consumer behavior: A study of eco-friendly hotel patrons. *Tour. Manag. Perspect.* 2019;31:171–180.

[ref35] Raina A Bathla G Mishra S : The role of ecotourism and environmental awareness in shaping travel behavior. *Examining Tourist Behaviors and Community Involvement in Destination Rejuvenation.* IGI Global Scientific Publishing;2024; pp.136–152.

[ref36] Rana VS Raina A Bathla G : The effect of sustainable practices on customer attitude: A study of sustainable hospitality operations. *Emirati Journal of Business, Economics, & Social Studies.* 2023;2(1):4–13. 10.54878/3er5qg34

[ref37] Shmueli G Sarstedt M Hair JF : Predictive model assessment in PLS-SEM: Guidelines for using PLSpredict. *Eur. J. Mark.* 2019;53(11):2322–2347. 10.1108/EJM-02-2019-0189

[ref38] Singh AK : a. Data set for the article titled “Do hotel green practices influence customer satisfaction? Evidence from the Indian hospitality sector”.[Data set]. *Zenodo.* 2025a. 10.5281/zenodo.17848960 PMC12805227 41551229

[ref39] Singh AK : b. Questionnaire.[Data set]. *Zenodo.* 2025b. 10.5281/zenodo.17848885

[ref40] Singh N Cranage DA Lee S : Green strategies for hotels: Estimation of recycling benefits. *Int. J. Hosp. Manag.* 2014;43:13–22. 10.1016/j.ijhm.2014.07.006

[ref41] Soni G Hussain S Kareem S : Environment friendly practices adopted in hotels and their impact on customer satisfaction: A critical review of the literature and research implications. *ATNA Journal of Tourism Studies.* 2022;17(1):115–142. 10.12727/ajts.27.5

[ref42] Yusof N Mohd Noor S Rahman A : Stakeholder perspectives on sustainable hospitality practices in Malaysia. *J. Clean. Prod.* 2022;356:131842. 10.1016/j.jclepro.2022.131842

[ref43] Zeithaml VA Berry LL Parasuraman A : The behavioral consequences of service quality. *J. Mark.* 1996;60(2):31–46. 10.1177/002224299606000203

