# Supplementary material for: Do hotel green practices influence customer satisfaction? Evidence from the Indian hospitality sector
Source: F1000Res. 2025 Dec 31;14:1480. [Version 1] doi: 10.12688/f1000research.175471.1 (PMC12805227; doi:10.12688/f1000research.175471.1)
Supplement: Peer review history

# Reviewer report: Reviewer response for version 1

**DOI:** 10.5256/f1000research.193456.r447179

**Published:** 2026-01-19

**Authors:** Tiurida Lily Anita

**Affiliations:** 1 Hotel Management, Bina Nusantara University - Joseph Wibowo Center Campus Library and Knowledge Center (Ringgold ID: 527027), West Jakarta, Jakarta, Indonesia

**Major revision.**

 The study is promising and the analysis framework is mostly appropriate, but the CS–CL direction inconsistency must be corrected, and replication/mediation reporting should be clarified for scientific soundness.1. The hypothesis/model direction inconsistency. The paper states H5: CS positively impact CL. But in the structural results and interpretation, it appears the tested path is CL **→ **CS (CL has “the strongest effect” on CS), and mediation logic treats CL as a mediator explaining CS.

Is the work clearly and accurately presented and does it cite the current literature?

Yes

If applicable, is the statistical analysis and its interpretation appropriate?

Partly

Are all the source data underlying the results available to ensure full reproducibility?

Partly

Is the study design appropriate and is the work technically sound?

Partly

Are the conclusions drawn adequately supported by the results?

Partly

Are sufficient details of methods and analysis provided to allow replication by others?

Partly

Reviewer Expertise:

Service Management, Hospitality Management, Hotel Management

I confirm that I have read this submission and believe that I have an appropriate level of expertise to confirm that it is of an acceptable scientific standard, however I have significant reservations, as outlined above.

# Reviewer report: Reviewer response for version 1

**DOI:** 10.5256/f1000research.193456.r449146

**Published:** 2026-01-14

**Authors:** Dr. Rajeev R Mishra

**Affiliations:** 1 A-Star Academy, Mumbai, India

"This manuscript investigates whether hotel green practices (HGP) influence customer satisfaction (CS) in the Indian hospitality sector, incorporating willingness to pay (WTP) and customer loyalty (CL) as mediating variables. The topic is timely and relevant, given the increasing importance of sustainability in hospitality and the evolving expectations of environmentally conscious consumers. However, the following points should be addressed to further strengthen the manuscript.

 1. Minor language editing: A light grammar and proofreading check would improve readability (e.g., consistent tense usage and minor spelling/word choice issues).

 2. Clarify sampling frame: Please ensure consistency in describing the sample (e.g., whether the study focuses only on five-star hotels or includes multiple star categories). This can be clarified in one line.

 3. Statistical reporting: Replace “p = 0.000” with the standard format “p < 0.001”.

 4. Common method bias statement: Since survey data is collected from a single source, adding one brief line about how common method bias was addressed/checked (e.g., full collinearity VIF or Harman single-factor test) would strengthen methodological transparency.

Is the work clearly and accurately presented and does it cite the current literature?

Yes

If applicable, is the statistical analysis and its interpretation appropriate?

Yes

Are all the source data underlying the results available to ensure full reproducibility?

Yes

Is the study design appropriate and is the work technically sound?

Yes

Are the conclusions drawn adequately supported by the results?

Yes

Are sufficient details of methods and analysis provided to allow replication by others?

No

Reviewer Expertise:

Hospitality/Tourism

I confirm that I have read this submission and believe that I have an appropriate level of expertise to confirm that it is of an acceptable scientific standard.

# Reviewer report: Reviewer response for version 1

**DOI:** 10.5256/f1000research.193456.r447175

**Published:** 2026-01-14

**Authors:** Varinder Singh Rana

**Affiliations:** 1 City University College of Ajman, Ajman, Ajman, United Arab Emirates

The study investigates the impact of Hotel Green Practices (HGP) on customers’ Willingness to Pay (WTP), Customer Loyalty (CL), and Customer Satisfaction (CS), integrating these constructs into a unified structural framework. While valuable, the article partly lacks clarity in sampling methods, measurement validity, and mediation analysis. To enhance scientific rigor, the authors should provide detailed justification of the sample, validate constructs with established scales, and clearly explain the statistical procedures for testing direct and mediated effects.

Is the work clearly and accurately presented and does it cite the current literature?

Yes

If applicable, is the statistical analysis and its interpretation appropriate?

Yes

Are all the source data underlying the results available to ensure full reproducibility?

Yes

Is the study design appropriate and is the work technically sound?

Yes

Are the conclusions drawn adequately supported by the results?

Yes

Are sufficient details of methods and analysis provided to allow replication by others?

Yes

Reviewer Expertise:

Hospitality Marketing, Hospitality Education, Sustainability, Ethics

I confirm that I have read this submission and believe that I have an appropriate level of expertise to confirm that it is of an acceptable scientific standard.

# Reviewer report: Reviewer response for version 1

**DOI:** 10.5256/f1000research.193456.r449140

**Published:** 2026-01-12

**Authors:** Dr. Neeraj Raj

**Affiliations:** 1 Lalit Narayan Mishra Institute of Economic Development and Social Change, Patna, India

The manuscript makes a meaningful contribution by demonstrating that green practices influence satisfaction largely through relational and value-based pathways (particularly customer loyalty as a strong mediator), offering valuable insights for hotel managers and policymakers in emerging markets such as India.

 Although the manuscript is publishable in its current form, the following minor refinements may further enhance clarity and presentation:

 1) Language and Grammar issue :

 A light proofreading is recommended to fix minor grammatical issues such as:

 •    “now a days” → “nowadays”

 •    “has become” vs “have become” agreement errors (e.g., “emissions… have become…”)

 These improvements will enhance readability without affecting content. 

 2) Sampling Description (Slight Clarification)

 The study reports data from “star-rated hotels of Delhi” and later states “only five star hotels in Delhi”. Please ensure consistency:

 •    If only five-star hotels were surveyed, mention this consistently in methodology and limitation section.

 •    If multiple star categories were included, specify which categories (3-star/4-star/5-star).

Is the work clearly and accurately presented and does it cite the current literature?

Yes

If applicable, is the statistical analysis and its interpretation appropriate?

Yes

Are all the source data underlying the results available to ensure full reproducibility?

Yes

Is the study design appropriate and is the work technically sound?

Yes

Are the conclusions drawn adequately supported by the results?

Yes

Are sufficient details of methods and analysis provided to allow replication by others?

Yes

Reviewer Expertise:

Management, Tourism And Hospitality

I confirm that I have read this submission and believe that I have an appropriate level of expertise to confirm that it is of an acceptable scientific standard.
